# Cardiology and the Cardiologist - Yesterday, Today and Tomorrow

**DOI:** 10.5935/abc.20190207

**Published:** 2019-09

**Authors:** Evandro Tinoco Mesquita, Aurea Lucia Alves de Azevedo Grippa de Souza

**Affiliations:** 1 Universidade Federal Fluminense, Niterói, RJ - Brazil; 2 Hospital Pró-Cardíaco, Rio de Janeiro, RJ - Brazil; 3 Diretoria de Qualidade Assistencial da Sociedade Brasileira de Cardiologia, Rio de Janeiro, RJ - Brazil

**Keywords:** Cardiology/history, Heart Diseases/history, History,19th Century, History,20th Century, Humans, Animals, Cardiology/trends, Brazilian Society of Cardiology/history

Carlos Chagas was the first “modern” cardiologist in Brazil.

Professor Nelson Botelho Reis

President of the SBC: 1945-1975 management

Cardiology as a medical specialty has been built from scientific knowledge derived from basic and clinical areas and from the development of technological devices that enabled us to study and understand the cardiovascular system. Two technological disruptive capacitors were: the stethoscope and the electrocardiogram, which allowed for the construction of two new “sciences”, the eletroctrocardiography and the phonomecanography. Certainly, the complexity of understanding the eletroctrocardiography and its electroclinical correlation has made Cardiology to become a specialty independent from clinical medicine, in the early 20th century. In the post-war years, technical-scientific developments allowed Cardiology to be prepared to become a solid area of action, and enabled the new search for therapeutic approaches, which contributed to the increased survival rates observed in basically all cardiopathies.

The foundation of the Brazilian Society of Cardiology (SBC), on August 14, 1943, was an important mark in our country, which enabled to tie together doctors dedicated to cardiovascular teaching, research and care. Thus, since 2005, the cardiologists day has been celebrated. The SBC and its brand logos have been transformed as it searches to connect our tradition and the contemporaneity of our cardiology and its international insertion and relevance (Figure 1).^1^

**Figure 1:**
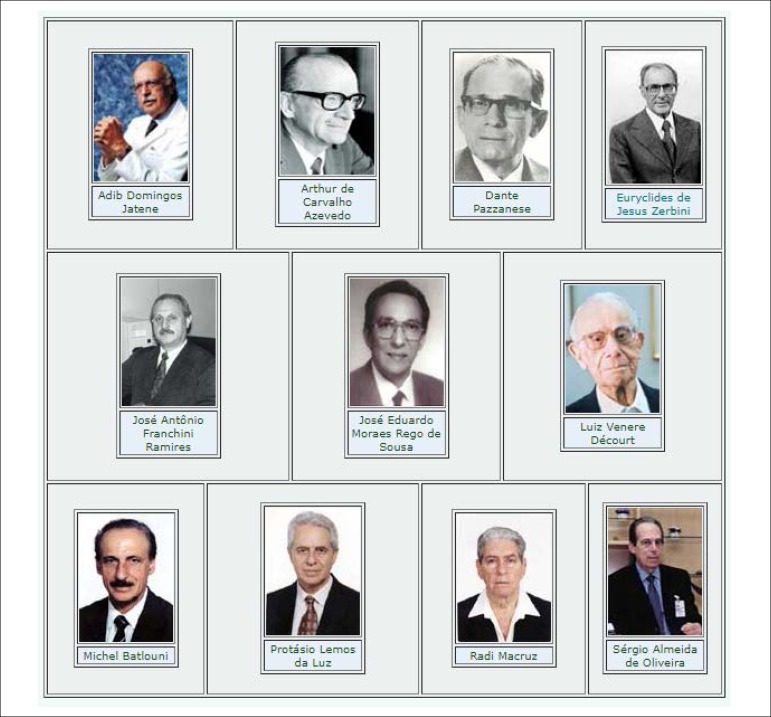
Brazilian cardiologists. Elected from SBC Partners as highlights of the 20^th^ century.^1^

The trajectory of this specialty traverses the emergence of the SBC, the propagation of training courses, the creation of the Arquivos Brasileiros de Cardiologia in 1948 and the first specialists’ titles. Over the years, in association with the hospitalist practice and the great technological development, Cardiology was divided into specific specialty areas: emergency cardiology and cardiac intensivism, congenital cardiopathies, cardiac surgery, hemodynamics, echocardiography, electrophysiology, among others. In order to understand the future, it is necessary to look back into the past and realize the huge steps of this specialty, which grows worldwide, aligned with the contemporaneity of the challenges to prevent and treat cardiovascular diseases in a safe manner, based on guidelines and focusing on the patient.

Going back to remote times, we can rescue the registries of the first steps of the specialty that we call protocardiology. Leonardo da Vinci’s observations, drawings and notes of the heart, made in 1490, are deemed as pioneering in the history of Cardiology. As author of the first graphic representation of the coronary arteries, he also explained the heart fluid dynamics and defined the formation of the aortic valve cusp and the sinus wall. It is possible to state that Da Vinci was a renaissance cardiologist.^2^ In the mid-1500s, Andreas Vesalius published a wonderful atlas of anatomy in his "Fabricius". In this publication, he called the heart the "center of life". This publication corrects the anatomical mistakes described by Galen and, for many historians, it is the mark of modern medicine. Modern cardiology has its beginnings with the publication of the book *De Motus Cordis*, written by the English physician and scientist William Harvey, the father of Cardiology,^3^ which caused a “hurricane” in science and medicine when it asserted, based on studies in animals and humans, that the blood goes from the heart through closed spaces and returns through veins into the heart.

The evolution of the history of Cardiology in Brazil was brilliantly revised by professor Nelson Botelho Reis and published in the *Arquivos Brasileiros de Cardiologia*, in 1986.^4^ He reinforces that the clinical-anatomic method was the first method in clinical medicine that evolved from a simple confirmation (“findings of necropsy”) or from the verification of the disease in an emerging scientific area, correlating the patient’s clinical picture. Important Italian (Malpigi, Morgami); French (Vienssens, Bichat and Laennec) and German (Virchow) physicians were fundamental to the construction of this new discipline. The Anatomopathology witnessed the emergence of Histology, which brought to light the importance of examining cells and tissues, and of expanding the correlation and causal mechanisms of diseases. The physicians interested in the area of Cardiology presented a growing and e robust method to explain the abnormal cardiovascular findings and necropsy data.

In our country, the central influence of the cardiovascular anatomoclinical method was important for the formation of cardiologists until the beginning of the 1980’s, initially influenced by Sylvio Carvalhal (São Paulo), Professor Luigi Bogliolo (Rio de Janeiro and Minas Gerais). Also, Professor Manoel Barreto Neto, whom I was honored to be a student of and from whom I received deep knowledge and the strength of the anatomoclinical method, along with the findings presented by Professor Raul Carlos Pareto Junior. The *Arquivos Brasileiros de Cardiologia* also publishes its traditional anatomoclinical section, an important tool for the formation of young cardiologists.

In the second half of the 19th century, the third stage of Cardiology emerged: the experimental medicine, the source of the brilliance of writers such as Claude Bernard and Carl Ludwig. The German and French schools made experimental medicine an independent area and two researchers described the Frank-Starling Law, the first law of the heart. In the 19th century, scientific advances derived from new disciplines such as microbiology, immunology, histology and biochemistry. Other sciences were incorporated into the study of Cardiology, such as physics and electricity, which contributed to the discovery of the x-ray and the chord galvanometer. In the future, these would constitute the basis of cardiac radiology and electrocardiography. In the end of the 19th century, the combination of anatomoclinical data and the recording of stetho-acoustic findings using graphic methods (phonomecanographic) consolidated a new pathophysiological approach, and a detailed description was made of a growing number of cardiovascular conditions.

It is possible to assert that the official study of Cardiology, as a science and medical specialty, started with the vital contribution of Dr. Carlos Chagas’ studies, who diagnosed the chagasic cardiomyopathy in 1909, and was responsible for the first translational studies and the publication of the first broad scientific study of a cardiac disease in Brazil. In addition, he was responsible for introducing the first electrocardiogram, installed in the laboratory of Manguinhos, a valuable and unequivocal contribution to Brazilian Cardiology.^4,5^

Industry development and migration from rural to urban areas, with the creation of metropolitan areas, in the early 1920s, led to a huge change in the eating and working habits of the Brazilian population, with the spread of heart diseases among the society.^6^ In the same period, the dissemination of the electrocardiograph made it possible to develop specific studies, which resulted in a branch of clinical medicine that became an area of major interest for numerous physicians, who began to search for specialty programs in Brazil and abroad. This movement, throughout the 1930s, led to the emergence of the medical specialty, thanks to the particular interest of this enthusiastic group for this specific study, which caused emerging resources to be redirected to the investigation of cardiovascular diseases, through phonomechanography and electrocardiography. From this moment, a great process of creation of annual specialty courses began, which initially were offered in São Paulo and Rio de Janeiro, even before the constitution of the Chair and the formation of this Society, on August 14, 1943. The access to these great transnational networks, and the whole American continent, benefited the investigation, the learning and trainings that took place until the end of World War II, and was the result of the internationalization process of the SBC, which maintained the possibility of interchange between Brazilian and North-American doctors and, consequently, of its actions together with the American College of Cardiology.^7^

Cardiology and cardiologists experienced deep changes in the beginning of the 20th century, due to the employment of the electrocardiogram as the cornerstone of cardiovascular rationale. Another important change was the progressive loss of relevance of the French school for Cardiology and the emergence of the North-American and Mexican schools of Cardiology. The Mexican Institute of Cardiology, founded by Ignacio Chaves, in 1944, became a research and education pole in the area of electrocardiography and a model center of cardiovascular education, attracting Brazilian young people for its residence in cardiology, as well as people from other nationalities. Between the 1940’s and 70’s, it would become a global reference center and contribute to important advances for the pathophysiology of congenital diseases, pulmonary hypertension, hemodynamics and electrophysiology. The influence of the North-American school starts to consolidate in the post-war period, when it incorporates economic resources to professionalize clinical research and construction and new centers of cardiology, as well as into the development of cardiac catheterization and myocardial revascularization surgery. The Harvard group led by Professor Eugene Braunwald consolidates in the discovery of new mechanisms of disease and in multicenter therapeutic studies.

In the 1980’s and 90’s, noninvasive cardiovascular imaging, echocardiography, myocardial scintigraphy, computerized tomography and cardiac resonance were incorporated and became increasingly indispensable for diagnostic, prognostic and therapeutic evaluation. Parallel to this, we started to incorporate the principles of evidence-based medicine into the process of clinical decision making in the therapeutic area. The Brazilian Society of Cardiology establishes clinical guidelines to guide the cardiovascular practice, incorporating and ranking the recommendations to confront several cardiovascular diseases.

Since the hospitalist practice of our cardiologists, cardiac surgery has been present and of vital importance for the development of our specialty, as well as hemodynamics, both being distinguished as the major departments in the first years of constitution of the SBC. Specialized sectors and equipment in hemodynamics and cardiac surgery, creation of specialized health care centers and units, such as the coronary unit and cardiac postoperative unit, provide specific care, adding knowledge and value to the specialty. With the arrival of the 1980's, there was the dissemination of the use of coronary angioplasty for the treatment of multiple vessel disease and acute myocardial infarction, using reperfusion techniques and thrombolytic therapy. The advances of interventionist hemodynamics through the use of pharmacological stents, valvuloplasty and prosthesis implantation emerged around the world, and quickly arrived in Brazil, promoting a huge evolution step towards the formation of the specialist in Cardiology, as well as the need for the constitution of teams dedicated to each area.^6-8^

All this movement promoted the growing and the development of the SBC, leading to the creation of the first five specialized Departments: Cardiovascular Surgery Department (1969), Pediatric Cardiovascular Department (1973), Cardiovascular and Respiratory Physiology Department (1974), Angiocardiography and Hemodynamic Department (1976) and Hypertension Department (1981).^4^ Nowadays, the SBC consists of 13 active departments, where a total of 14,000 associates are distributed in 26 regional clusters throughout Brazil, being the largest society in Latin America. Cardiology, in addition to having being the first specialty to establish a Chair at universities and teaching hospitals, was also responsible for the emergence of accreditation procedures for specialist titles and, nowadays, has the support of 8,429 doctors with a specialty degree in Cardiology.

In addition, several institutions offer *lato sensu* post-graduation courses. In line with this demand for specialty courses, the SCB, alongside its regular publications *Arquivos Brasileiros de Cardiologia* (Brazilian Archives of Cardiology), *International Journal of Cardiovascular Science, the SBC Journal* and its guidelines, provides DE (distance education) courses, a broad schedule of events and congresses with online access. In order to respond the need for expanding translational research, the specialty counts with the possibility of network research, as in the case of stem-cell therapy, which involved several centers, the support to clinical trials and registries, in addition to epidemiologic studies on the most prevalent cardiovascular conditions in our field. In the context of standardization of conduct and creation of protocols, the SBC dedicates extreme determination for the development and publication of guidelines on approach and treatment of the main diseases with high morbidity and mortality nowadays.

In the late 20th century, Brazilian Cardiology constructed a solid legacy and international relevance, a trajectory from which renowned fellows were elected representatives of this first century of our specialty (Figure 2).

**Figure 2:**
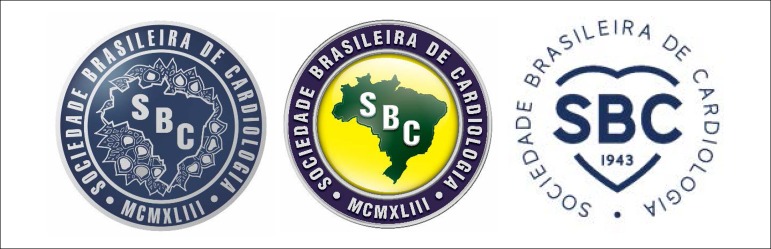
Different SBC logos throughout its history.

This new decade emerges with a new design for Cardiology in a digital, connected, patient-focused environment, based on major pillars - artificial intelligence, big-data, robotics, biosensors, telemedicine, devices, sensors and genomics. Together they will support the Precision Cardiology paradigm. The need to incorporate into our mindset the idea of the cardiologist approaching the world of innovation and entrepreneurship is a new challenge in the formation of our new cardiologists with emphasis on the development of female leadership in cardiology. From the assistance point of view, our integration with the population health and family doctors will increase, as well as our multidisciplinary approaches, enabling the implementation of lines of care and the monitoring of clinical outcomes and focused on patient safety. This means to know and dominate the triad formed by cardiovascular biomedical knowledge, technology (Digital medicine) and humanization. Knowledge moves forward quickly and unstoppable, in a global manner, supported and favored by numerous opportunities of knowledge exchange between peers and institutions worldwide. The technology, which promotes the evolution of Digital and Precision Medicine, favors the access to knowledge, the strengthening of diagnosis, the promotion of man-machine interaction and the possibility of personalizing therapy to its molecular level. And, finally, the rescue of essence: the humanization of such advanced and futuristic Cardiology. A new journey will be necessary into the daily practice of doctor-patient interaction in order to promote empathy, patient empowerment and sensitivity to the doctor inserted in this new context for this new decade.^9-13^

## References

[1] Sociedade Brasileira de Cardiologia Cardiologistas em destaque. Eleitos a partir dos Sócios a
SBC(2002).

[2] Cambiaghi M, Hausse H (2019). Leonardo da Vinci and his study of the Heart: A 500-year
Anniversary appreciation of a maestro. Eur Heart J.

[3] Reichert P A History of the Development of Cardiology as a Medical
Specialty.

[4] Reis NB (1986). Evolução Histórica da Cardiologia no
Brasil. Arq Bras Cardiol.

[5] Albanesi Fº FM (2005). 50 anos de história da cardiologia do Estado do Rio de
Janeiro.

[6] Geison GL, Foster M, Cambridge School of Physiology (1985). The emergence of modern cardiology. Med Hist Suppl.

[7] Souza ROP (2017). História da cardiologia no Brasil: a construção de
uma especialidade médica (1937-1958).

[8] Braunwald E (2014). The Ten Advances That Defined Modern Cardiology. Trends Cardiovasc Med.

[9] Rao GRH (2018). Modern Day Cardiology: Expectations and
Limitations. J Cardiol.

[10] Califf RM (2018). Future of personalized Cardiovascular Medicine. JACC.

[11] Evans J, Banerjee A (2016). Global health and data science: future needs for tomorrow's
cardiologista. Br J Cardiol.

[12] Mountford J (2018). What do tomorrow's doctors really need to know?. BMJ Leader.

[13] Brush JE (2018). Is the Cognitive Cardiologist Obsolete?. JAMA Cardiol.

